# Everolimus-Eluting Biodegradable Abluminal Coating Stent versus Durable Conformal Coating Stent: Termination of the Inflammatory Response Associated with Neointimal Healing in a Porcine Coronary Model

**DOI:** 10.1155/2020/1956015

**Published:** 2020-04-27

**Authors:** Masayuki Mori, Kenji Sakata, Junichiro Yokawa, Chiaki Nakanishi, Kota Murai, Hirofumi Okada, Masaya Shimojima, Shohei Yoshida, Kazuaki Yoshioka, Yoh Takuwa, Kenshi Hayashi, Masakazu Yamagishi, Masa-aki Kawashiri

**Affiliations:** ^1^Department of Cardiovascular and Internal Medicine, Kanazawa University Graduate School of Medicine, Kanazawa, Japan; ^2^Department of Molecular Vascular Physiology, Kanazawa University Graduate School of Medicine, Kanazawa, Japan; ^3^Osaka University of Human Sciences, Osaka, Japan

## Abstract

**Objectives:**

We evaluated the effect of the different carrier systems on early vascular response through histological analysis and scanning electron microscopy using a porcine model.

**Background:**

Although Synergy™ and Promus PREMIER™ share an identical stent material and drug elution (everolimus), they use different drug carrier systems: biodegradable abluminal coating polymer or durable conformal coating polymer, respectively. However, data regarding the impact of the different coating systems on vessel healing are currently limited.

**Methods:**

Twelve Synergy™ and Promus PREMIER™ were implanted in 12 swine. Histopathological analysis of the stented segments was performed on the 2^nd^ and 14^th^ days after implantation. Morphometric analysis of the inflammation and intimal fibrin content was also performed.

**Results:**

On the 2^nd^ day, neointimal thickness, percentage of neointimal area, and inflammatory and intimal fibrin content scores were not significantly different between the two groups. On the 14^th^ day, the inflammatory and intimal fibrin content scores were significantly lower in Synergy™ versus those observed in Promus PREMIER™. In Synergy™, smooth muscle cells were found and the neointimal layers were smooth. In contrast, inflammatory cells were observed surrounding the struts of Promus PREMIER™.

**Conclusions:**

These results demonstrate that termination of reactive inflammation is accelerated after abluminal coating stent versus implantation of conformal coating stent.

## 1. Introduction

Drug-eluting stents (DES) were developed for reduction of in-stent restenosis after implantation of stents [1, 2]. Consequently, DES have become the mainstay for coronary intervention in coronary artery disease, regardless of the size of the coronary arteries [3]. However, DES have been associated with delayed vascular healing, which may lead to the occurrence of late complications [4–6]. Local reaction to the polymer coating may exacerbate the inflammatory reaction caused by the implantation of stent [7]. Thus, delayed endothelialization of the stent struts and positive vessel remodeling may occur, resulting in increased rates of late complications. Therefore, it is important to examine the effects of the stent coating systems on early vascular response.

Synergy™ and Promus PREMIER™ (Boston Scientific Corporation, Marlborough, MA, USA) have been made of the same material (but the structure is different) and eluting drug (i.e., everolimus). However, they use different drug carrier systems: biodegradable abluminal coating polymer or durable conformal coating polymer. We previously reported that stents with abluminal coating result in early neointimal healing and less inflammatory reaction [8]. This results in reduction of polymer exposure due to the complete absorption of the polymer shortly after termination of drug elution and absence of the polymer on the luminal side. Clinical evidence has demonstrated the advantage of the stent with abluminal coating in terms of long-term prognosis [9].

The long-term studies already showed the safety and efficacy of abluminal coating stent using a porcine coronary model [10]. However, data regarding the impact of the different coating systems (i.e., abluminal or conformal) on vessel healing are currently limited. The present study evaluated the impact of the different carrier systems of everolimus-eluting stents on early vascular response through histological analysis and scanning electron microscopy (SEM) using a porcine model.

## 2. Materials and Methods

### 2.1. Animal Study Protocol

The protocol of this animal study was approved by the Animal Care and Use Committee of Kanazawa University. The experiments were conducted according to the “Basic Guidelines for Conduct of Animal Experiments” published by the Ministry of Health, Labour and Welfare, Japan.

Twelve Synergy™, everolimus-eluting stent with biodegradable abluminal coating polymer (poly-lactide-co-glycide (PLGA)) (length: 12 mm, diameter: 3.0 mm) and 12 Promus PREMIER™, everolimus-eluting stent with durable conformal coating polymer (polyvinylidene difluoride (PVDF)) (length: 12 mm, diameter: 3.0 mm) were implanted in noninjured coronary arteries of 12 domestic swine (female, 3-4 months old). Both types of stents are manufactured using platinum chromium alloy.

All swine were treated with aspirin (200 mg, Bayer, Land Nordrheine-Westfalen, Germany) and clopidogrel (300 mg, Sanofi Aventis, Gouda, The Netherlands) preinterventionally. Moreover, aspirin (200 mg/day) and clopidogrel (75 mg/day) were administered daily until the end of the study. Following the induction of anesthesia with intramuscular administration of ketamine (20 mg/kg), the animals were maintained under general anesthesia using sevoflurane and oxygen. During the intervention, we carefully observed the level of anesthesia to maintain appropriate sedation. The electrocardiogram and heart rates of the animals were continuously monitored using a polygraph recording system (OptiPlex755, Nihon-Kohden, Tokyo, Japan) throughout the entire procedure. Heparin was the 5000 IU administered at the beginning via the left carotid artery using a sheath, followed by injection of 2000 IU per hour.

Stent deployment was performed as previously described [11, 12]. Vessel allocation to experimental groups was distributed into the different stent types equally in three different coronary arteries. Each animal received Synergy™ and Promus PREMIER™ in the coronary arteries. A single stent was implanted in each coronary artery. Stent deployment was performed using a 1 : 1.1-1.2 stent-to-artery diameter ratio. However, if deemed necessary, we performed balloon dilation to optimize coronary stenting. We followed swine until the 2^nd^ (*n* = 6) or 14^th^ (*n* = 6) day after implantation. At the end of the study, the animals were sacrificed under general anesthesia, and the hearts were extracted and examined through SEM and histological analysis. Under these conditions, we controlled anesthesia not to response animal pain.

### 2.2. Tissue Preparation

Twenty stented vessels (*n* = 5, each stent and group) were perfused with saline, perfusion-fixed with 4% formaldehyde, and embedded in GMA resin and N, N-dimethyl aniline. Sections (three sections per stent, 5-6 *µ*m thick; two is using for hematoxylin and eosin and one is using for immunostaining) were cut using a cemented tungsten carbide knife (RM2245, Leica, Germany), and stained with hematoxylin and eosin (New Histology Science Laboratory Corporation, Tokyo, Japan). The sections were subsequently evaluated using an optical microscope (BZ-9000, KEYENCE, Osaka, Japan).

The presence of smooth muscle cells was evaluated through immunostaining (New Histology Science Laboratory Corporation, Tokyo, Japan). After deparaffinization, the stented sections performed antigen activation (heat treatment under alkaline condition) after deparaffinized. Thereafter, the sections were incubated with 0.3% hydrogen peroxide solution and labelled with an anti-actin smooth muscle mouse monoclonal antibody (AN-128-5M, BioGenex laboratories, Fremont, CA, USA), followed by One-Step Polymer-HRP (HK595-50K, BioGenex laboratories). The sections were incubated with the DAB reagent, and counter staining was performed using Mayer's Hematoxylin Solution. Immunohistological images were evaluated using the BZ-9000 optical microscope. The presence of smooth muscle cells and fibrin was also evaluated trough fluorescent immunostaining (Supplementary method).

For SEM, 4 stented vessels (*n* = 1, each stent and group) were fixed with 2.5% glutaraldehyde containing PBS for 2 hours, treated with a series of dehydration steps using graded ethanolic aqueous solutions (50, 70, 90, 99, 99, 99% ethanol, 10 minutes per step), freeze-dried in t-butyl alcohol, and sputter-coated with platinum. SEM images were obtained using the JSM-5400 system (JEOL, Nagoya, Japan) at an acceleration voltage of 10 kV.

### 2.3. Histological Analysis

Histological evaluation included the measurement of neointimal thickness and percentage of neointimal area. Neointimal thickness was measured above the stent and over the stent struts (each mid-portion) and averaged in that cross-section. Neointimal areas were measured around the stent and above the inner membrane. % neointimal area was defined as 100 × neointimal area/stent area [13]. To consider the different stent structure, we have measured strut cross-sectional area (StrCSA: strut width × its thickness) [14] and introduced the parameters, neointimal thickness/StrCSA and % neointimal area/StrCSA (% neointimal area/StrCSA × 16: the number of struts). We could not obtain the data of both stent struts' widths (confidential information); we have measured them ourselves (Synergy™ was 86 *μ*m and its StrCSA was 6794 *μ*m^2^, Promus PREMIER™ was 67 *μ*m and its StrCSA was 5427 *μ*m^2^). Morphometric analysis of inflammation, injury (graded from 0 to 3), and intimal fibrin content scores (graded from 1 to 3) was also performed. These parameters were calculated as previously described [15, 16]. The histological parameters were measured via digital morphometry.

### 2.4. Statistical Analysis

All data are expressed as mean ± standard deviation. The parameters (neointimal thickness, % neointimal area, neointimal thickness/StrCSA, % neointimal area/StrCSA, and inflammatory score, fibrin content, and injury scores) were compared using the nonparametric Wilcoxon rank-sum test for 2^nd^ day and 14^th^ day for each group. A *P* value < 0.05 was considered statistically significant (JMP software, SAS Institute Inc., Cary, NC, USA).

## 3. Results

Synergy™ and Promus PREMIER™ were successfully implanted in the coronary arteries of 12 swine. All animals survived the procedure and remained healthy until the end of the study.

### 3.1. Neointimal Coverage and Healing

**Figure 1:**
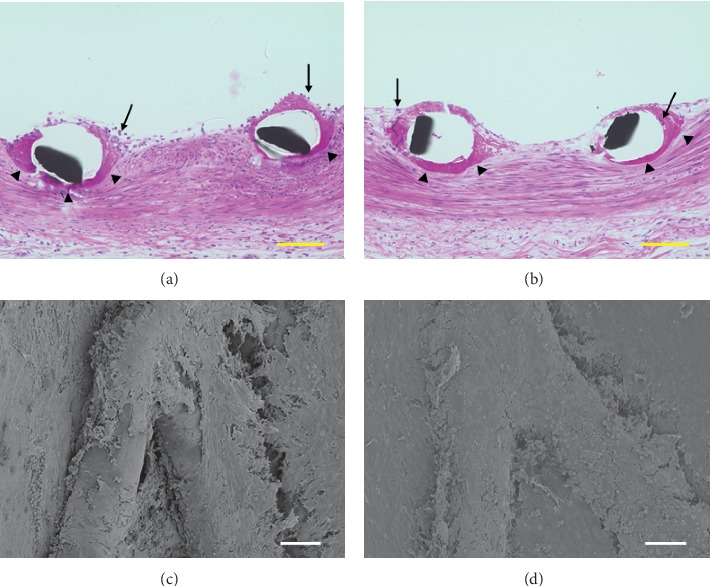
Representative histological and electron microscopic images of Promus PREMIER™ and Synergy™ on the 2^nd^ day after implantation. Promus PREMIER™ (a) and Synergy™ (b) were partially covered with neointima which mainly contained abundant fibrin (black arrow head). Inflammatory cells, such as monocytes and blood cells, were aggregated, surrounding the struts of both stents (black arrow). As observed through scanning electron microscopy, Promus PREMIER™ (c) and Synergy™ (d) were partially covered with a thin tissue resembling the neointima, although their surfaces were still coarse. The scale bars are 100 *µ*m (histological images) and 30 *µ*m (electron microscopic images).

On the 2^nd^ day, histological images showed that Synergy™ and Promus PREMIER™ were partially covered with neointima, mainly containing abundant fibrin (Figures 1(a) and 1(b)). In addition, spindle and round cells, as well as inflammatory cells such as monocytes and blood cells, were present, surrounding the struts of both stents. Although the surfaces of the stents appeared to be coarse, SEM images showed that both types of stents were partially covered with a thin tissue resembling the neointima (Figures 1(c) and 1(d)).

**Figure 2:**
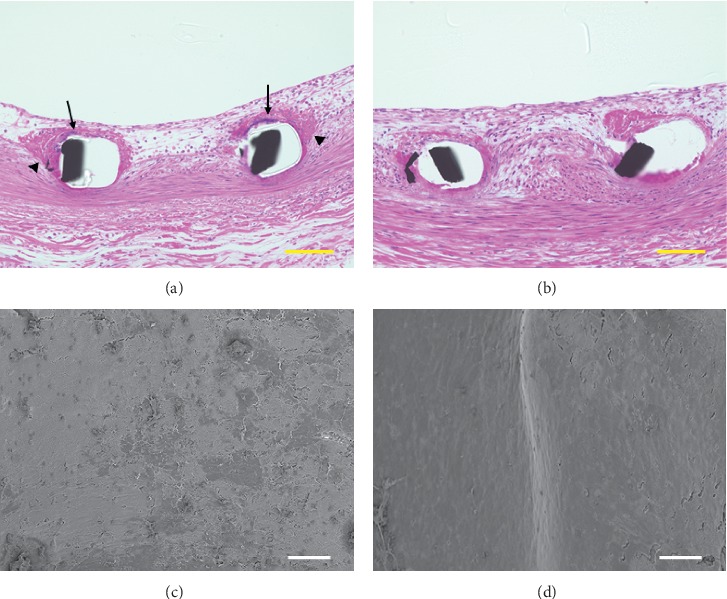
Representative histological and electron microscopic images of Promus PREMIER™ and Synergy™ on the 14^th^ day after implantation. Promus PREMIER™ (a) and Synergy™ (b) were completely coved with the neointimal layer. The struts of Promus PREMIER™ showed moderate deposition of fibrin (black allow head) and aggregation of inflammatory cells (black allow). Smooth muscle cells were present in Synergy™. As observed through scanning electron microscopy, Promus PREMIER™ (c) and Synergy™ (d) were completely covered with the neointimal layer with smoother surface than that observed on the 2^nd^ day. The scale bars are 100 *µ*m (histological images) and 30 *µ*m (electron microscopic images).

**Figure 3:**
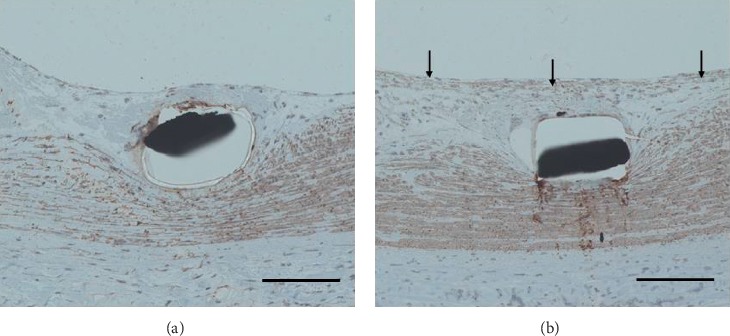
Representative immunohistological images of Promus PREMIER™ and Synergy™ on the 14^th^ day after implantation. Staining showed that in both Promus PREMIER™ (a) and Synergy™ (b), the smooth muscle tissue of the original parts was positive for antiactin, smooth muscle antibody. In particular, smooth muscle cells were mainly present at the luminal side of the neointimal layer in Synergy™ ((b), black arrow). The scale bar is 100 *µ*m.

On the 14^th^ day, histological images showed that Synergy™ and Promus PREMIER™ were completely covered with neointimal layer (Figures 2(a) and 2(b)). Promus PREMIER™ showed moderate deposition of fibrin and aggregation of inflammatory cells, although these were observed to be few in Synergy™. Interestingly, smooth muscle cells, which were prominently present in Synergy™ stents, were rarely found in Promus PREMIER™ stents. Staining showed that these cells were immunohistologically positive for anti-actin smooth muscle antibody (Figures 3(a) and 3(b)). Smooth muscle cells were observed mainly at the luminal side of the neointimal layer (Supplementary figure). SEM images showed that both stents were completely covered with the neointimal layer, with smoother surfaces than those observed on the 2^nd^ day (Figures 2(c) and 2(d)).

### 3.2. Histological Analysis

**Figure 4:**
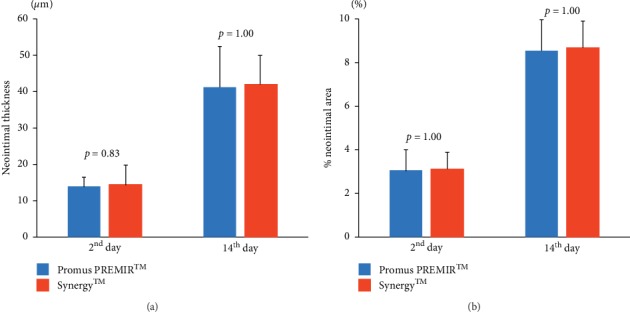
Quantitative analysis of neointimal thickness and % neointimal area. There were no significant differences in neointimal thickness (a) and % neointimal area (b) between Promus PREMIER™ (*n* = 5) and Synergy™ (*n* = 5) on the 2^nd^ and 14^th^ days.

**Figure 5:**
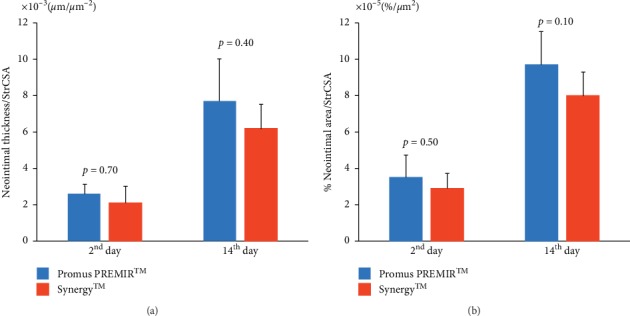
Quantitative analysis of neointimal thickness/StrCSA and % neointimal area/StrCSA. There were no significant differences in neointimal thickness/StrCSA (a) and % neointimal area/StrCSA (b) between Promus PREMIER™ (*n* = 5) and Synergy™ (*n* = 5) on the 2^nd^ day and 14^th^ day. StrCSA: strut cross-sectional area.

There were no significant differences in the neointimal thickness and % neointimal area between Promus PREMIER™ (*n* = 5) and Synergy™ (*n* = 5) on the 2^nd^ day (13.9 ± 2.7 *µ*m vs. 14.4 ± 5.4 *µ*m, *P*=0.83 and 3.1 ± 0.9% vs. 3.1 ± 0.7%, *P*=1.00, resp.) (Figures 4(a) and 4(b)). On the 14^th^ day, the neointimal thickness and % neointimal area of Promus PREMIER™ (*n* = 5) and Synergy™ (*n* = 5) were 41.6 ± 10.9 *µ*m vs. 42.4 ± 7.8 *µ*m (*P*=1.00) and 8.5 ± 1.4% vs. 8.7 ± 1.2% (*P*=1.00), respectively (Figures 4(a) and 4(b)). In addition, there were no significant differences in neointimal thickness/StrCSA and % neointimal area/StrCSA between Promus PREMIER™ and Synergy™ on the 2^nd^ day (2.6 × 10^−3^ ± 0.5 × 10^−3^ *μ*m/*μ*m^2^ vs. 2.1 × 10^−3^ ± 0.9 × 10^−3^ *μ*m/*μ*m^2^, *P*=0.70 and 3.5 × 10^−5^ ± 1.2 × 10^−5^%/*μ*m^2^ vs. 2.9 × 10^−5^ ± 0.8 × 10^−5^%/*μ*m^2^, *P*=0.50, resp.) and 14^th^ day (7.7 × 10^−3^ ± 2.3 × 10^−3^ *μ*m/*μ*m^2^ vs. 6.2 × 10^−3^ ± 1.3 × 10^−3^ *μ*m/*μ*m^2^, *P*=0.40 and 9.7 × 10^−5^ ± 1.8 × 10^−5^%/*μ*m^2^ vs. 8.0 × 10^−5^ ± 1.3 × 10^−5^%/*μ*m^2^, *P*=0.10, resp.) (Figures 5(a) and 5(b)).

**Figure 6:**
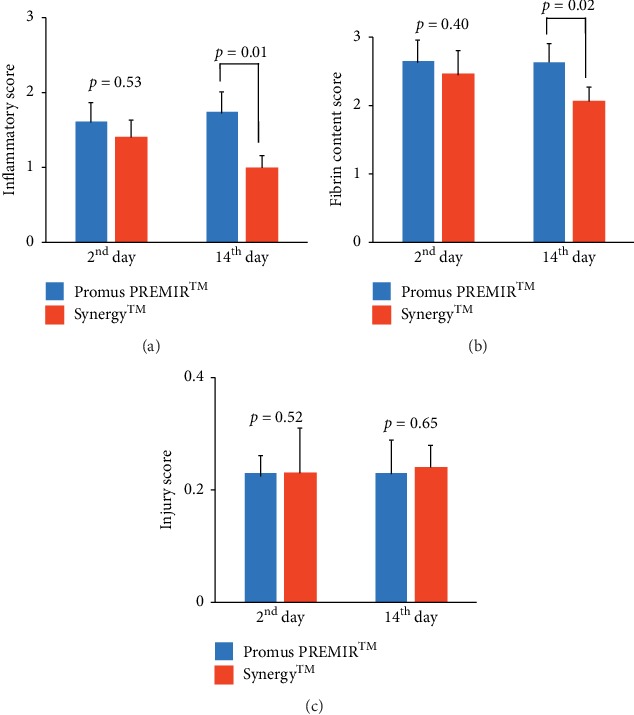
Quantitative analysis of the inflammatory, fibrin content and injury scores. On the 2^nd^ day, there were no significant differences between Promus PREMIER™ (*n* = 5) and Synergy ™ (*n* = 5) in the inflammatory score (*P*=0.53), fibrin content score (*P*=0.40), and injury (*P*=0.52) score. On the 14^th^ day, inflammatory score (*P*=0.01) and fibrin content score (*P*=0.02) were significantly lower in Synergy™ (*n* = 5) than in Promus PREMIER™ (*n* = 5), although the injury score (*P*=0.65) was not different between the two groups.

Moreover, on the 2^nd^ day, there were no significant differences in inflammatory (1.61 ± 0.26 vs. 1.40 ± 0.23, *P* = 0.53), fibrin content (2.65 ± 0.31 vs. 2.46 ± 0.35, *P* = 0.40), and injury (0.23 ± 0.08 vs. 0.23 ± 0.03, *P* = 0.52) scores between the two groups (*n* = 5, each type of stent) (Figures 6(a)–6(c). On the 14^th^ day, there was no significant difference in injury scores (0.23 ± 0.06 vs. 0.24 ± 0.04, *P* = 0.65) between the two groups. However, inflammatory (1.74 ± 0.27 vs. 0.99 ± 0.17, *P* = 0.01) and fibrin content (2.63 ± 0.27 vs. 2.06 ± 0.21, *P* = 0.02) scores were significantly lower in Synergy™ (*n* = 5) than those in Promus PREMIER™ (*n* = 5) (Figures 6(a)–6(c)). Although there was no difference observed in neointimal proliferation, these results demonstrated that the use of Synergy™ may accelerated the termination of inflammatory response compared with Promus PREMIER™.

## 4. Discussion

The findings of the present study showed that stent with abluminal coating system induced early neointimal healing with less inflammatory reaction compared with that observed in stent with conformal coating system. Although the development of neointima was not different between the two groups within 14 days after implantation, this finding suggests the involvement of a mechanism linked to early neointimal healing with less inflammatory reaction after implantation of stents with abluminal coating system.

The neointimal healing response after drug-eluting stent implantation is affected by many factors. There is not only the drug type and coating system but also the stent platform (its structure and material) [14]. Synergy™ and Promus PREMIER™ share an identical stent material and drug elution (everolimus, 1 *µ*g/mm^2^) but they use different drug carrier systems (bioresorbable abluminal coating polymer or durable conformal coating polymer) and stent structure. The bioabsorption of PLGA is initiated immediately after implantation; however, the greatest loss of PLGA mass has been observed between days 30 and 90. In the present study, 14 days after the intervention, the PLGA mass remained the same as the PVDF durable polymer. In addition, during 14 days, the dose of everolimus and rate of drug elution were similar between the two types of stents (data from Boston Scientific Japan). Regarding the difference in both stent structures, we have evaluated neointimal proliferation using StrCSA. Although neointimal proliferation is affected by stent strut's geometry [14], the neointimal parameters using StrCSA of our study were not significantly different between two types of stents on the 2^nd^ and 14^th^ day. We guess that neointimal proliferation might be less affected by stent structure because our experimental period was short. Previous report suggested that the larger StrCSA showed the bigger neointimal burden [14]. In our study, Synergy™ with abluminal coating polymer showed larger StrCSA than Promus PREMIER™ with durable conformal coating polymer. From these findings, the abluminal coating polymer might be associated with the reduction of neointimal burden.

On the 2^nd^ day, the inflammatory reaction (inflammatory and fibrin content score) was similar between Synergy™ and Promus PREMIER™. However, on the 14^th^ day, these parameters were significantly lower in Synergy™ versus those observed in Promus PREMIER™. The neointimal thickness and % neointimal area were similar between Synergy™ and Promus PREMIER™ on the 2^nd^ and 14^th^ days. In addition, histological and SEM images showed similar neointimal coverage above the struts of Synergy™ and Promus PREMIER™ despite the different inflammatory response observed between the two groups. The histological results indicated less inflammatory response associated with Synergy™ compared with that observed with Promus PREMIER™. It is possible that less inflammatory response was due to abluminal coating system. The polymer itself causes inflammatory response, as a biological reaction [7, 17]. Abluminal coating polymer is applied only on vessel side; on the other hand, conformal coating polymer is applied around stent. Conformal coating stent has larger area of polymer compared to abluminal coating stent. Therefore, there is a possibility that inflammatory response is prolonged and strong in conformal coating stent. Importantly, smooth muscle cells, which were prominently observed in Synergy™ stents, were rarely found in Promus PREMIER™ stents. This result suggests that less inflammatory response could induce smooth muscle cell at early phase after stent implantation.

The everolimus-eluting abluminal coating stent has been shown to be safe and efficacious in coronary artery intervention [9, 18, 19]. Biodegradable abluminal coating polymers, as opposed to durable conformal coating polymers, offer advantages in coronary stent technology such as complete drug elution and reduced inflammatory response, potentially decreasing the risk of late complications [20]. Our present experimental data showed that abluminal coating stent induces neointimal healing with less inflammatory reaction at early phase after implantation. This result suggests that the abluminal coating system accelerates the termination of the inflammatory response in the vessel wall. The previous study demonstrated that the response to healing after placement of the coronary stent in a human coronary artery is 5-6 times longer than in a porcine coronary artery [21]. Therefore, our experimental data at 14 days after this two-stent implantation in swine may correspond to a reasonable approximation of 3 months in humans. In clinical setting, early endothelialization has the potential to reduce dual antiplatelet therapy period especially in patients with high bleeding risk. Moreover, previous studies have demonstrated the advantage of abluminal coating stent in terms of long-term prognosis [9, 22]. Taken together, abluminal coating stent has the clinical advantage compared to conformal coating stent from the point of early neointimal healing with less inflammatory response.

## 5. Limitations

The present study was characterized by limitations. Firstly, only healthy atherosclerosis-free animals were used in these experiments. Therefore, the current findings should be interpreted with caution when extrapolating to arteriosclerotic disease. Secondly, the study included only two time points (the 2^nd^ and 14^th^ days) after implantation of the stents. At the end of the study, we observed higher inflammatory response associated with the conformal coating stent than that observed with the abluminal coating stent. Although long-term studies (3 to 6 months after implantation) have shown minimal inflammatory reaction in abluminal coating stent compared with that observed in conformal coating stent [23], the duration of this difference remains unclear.

## 6. Conclusions

The present results demonstrated that termination of reactive inflammation is accelerated after implantation of abluminal coating stent versus implantation of conformal coating stent. Although further clinical investigation is necessary to confirm these results, the abluminal coating stent appears to offer a clinical advantage in terms of early neointimal healing with less inflammatory response.

## Data Availability

The data used to support the findings of this study are included within the article.

## Supplementary Materials

Supplementary Materials Representative fluorescent immunostaining images of Promus PREMIER™ and Synergy™ on the 14^th^ day after implantation. The smooth muscle tissue was stained positive for anti-alpha smooth muscle actin and fibrin was stained positive for anti-Human Fibrinogen. In Synergy™, there were many smooth muscle tissues at the luminal side of the neointimal layer compared with Promus PREMIER™. The scale bar is 20 *µ*m.
