# Supplementary material for: Everolimus-Eluting Biodegradable Abluminal Coating Stent versus Durable Conformal Coating Stent: Termination of the Inflammatory Response Associated with Neointimal Healing in a Porcine Coronary Model
Source: J Interv Cardiol. 2020 Apr 27;2020:1956015. doi: 10.1155/2020/1956015 (PMC7201493; doi:10.1155/2020/1956015)
Supplement: Supplementary Materials — Representative fluorescent immunostaining images of Promus PREMIER™ and Synergy™ on the 14th day after implantation. The smooth muscle tissue was stained positive for anti-alpha smooth muscle actin and fibrin was stained positive for anti-Human Fibrinogen. In Synergy™, there were many smooth muscle tissues at the luminal side of the neointimal layer compared with Promus PREMIER™. The scale bar is 20 µm. [file 1956015.f1.pdf]

Supplementary MATERIALS AND METHODS

Tissue Preparation

The presence of smooth muscle cells and fibrin were evaluated trough fluorescent immunostaining. The stented sections were rinsed 3 times with phosphate buffered saline (PBS) and treated with target retrieval solution (S2031, Dako, Tokyo, Japan) according to manufacturer's instructions. The sections were maintained at a sub-boiling temperature (30 min). Thereafter, the sections were cooled on the bench (30 min) and incubated in 0.3% TritonX-100 in PBS (30 min). The sections were rinsed PBS (3 min × 2) and incubated in 5% normal goat serum blocking solution (143-06561, Wako, Osaka, Japan) / 0.3% TritonX-100 in PBS (30 min). The sections were labeled with anti-α Smooth Muscle Actin (A2547, SIGMA, Saint Louis, MO, USA) at a dilution ratio of 1:400, followed by Alexa Fluor 568-conjugated anti-mouse IgG2a antibody (A-21134, ThermoFisher SCIENTIFIC, Rockford, IL, USA) at a dilution ratio of 1:2000 and anti-Human Fibrinogen (A0080, Dako, Tokyo, Japan) at a dilution ratio of 1:400, followed by Alexa Fluor 647-conjugated anti-rabbit IgG antibody (A27040, ThermoFisher SCIENTIFIC, Rockford, IL, USA) at a dilution ratio of 1:1000. Nuclei were stained with DAPI (D1306, Molecular

Probes, Life Technologies, Carlsbad, CA, USA).

Immunofluorescence images were evaluated using confocal microscopy was carried out on an inverted Nikon Eclipse Ti2 confocal microscope (Nikon Instruments/Nikon Corp., Tokyo, Japan) equipped with an Andor Dragonfly spinning-disk unit, Andor EMCCD camera (iXon DU888; Andor Technology Ltd.) and a laser unit (Coherent Inc., Santa Clara, CA, USA). An oil-immersion objective (PlanApo 60X; NA 1.4; Nikon Corp.) was used for all experiments. Excitation for DAPI, Alexa 568 and Alexa 647 chromophores were provided by a 405-, 561- and 637-nm laser, respectively.

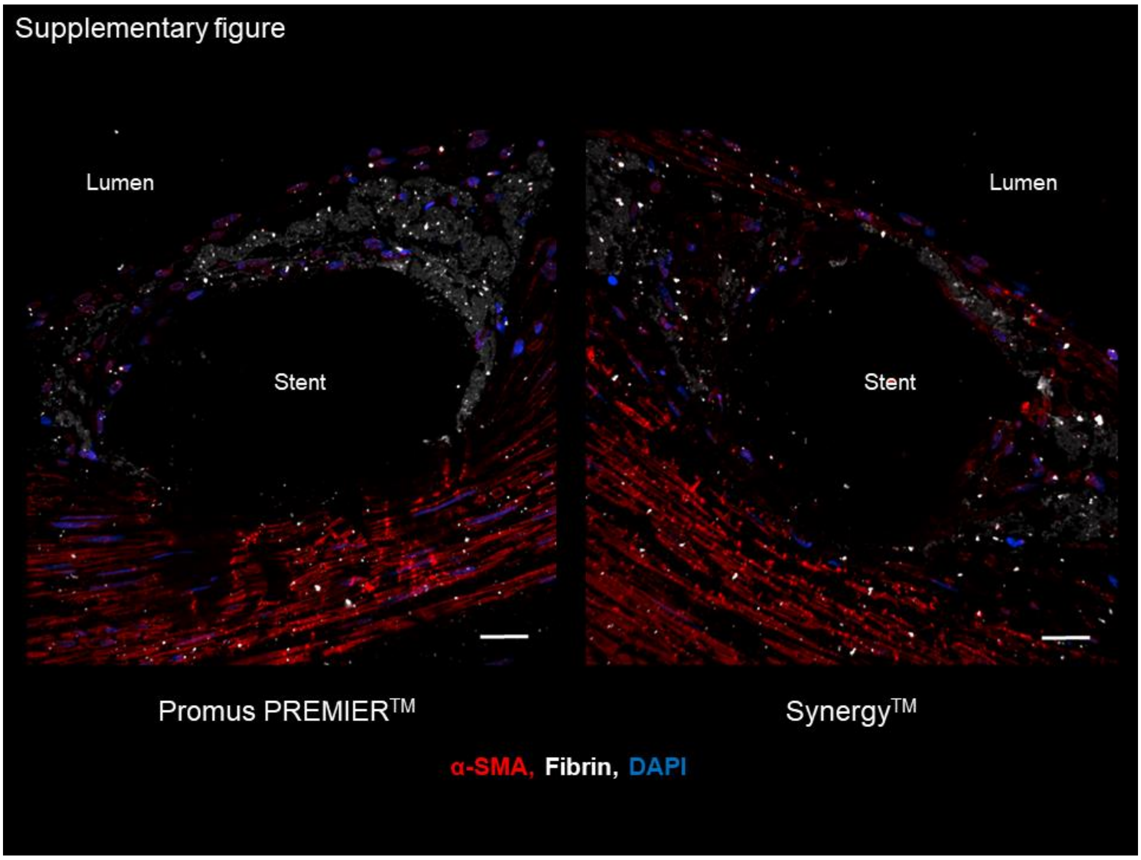

SUPPLEMENTARY FIGURE. Representative fluorescent immunostaining images of Promus PREMIER™ and Synergy™ on the 14th day after implantation. The smooth muscle tissue was stained positive for anti- α Smooth Muscle Actin and fibrin was stained positive for anti-Human Fibrinogen. In Synergy™, there were many smooth muscle tissue at the luminal side of the neointimal layer compared with Promus PREMIER™. The scale bar is 20 μm.
